# High‐intensity fire and long‐term destocking causes top kill but fails to reverse encroachment and restore biodiversity

**DOI:** 10.1002/eap.70341

**Published:** 2026-10-22

**Authors:** Kalea R. Nippert‐Churchman, Zak Ratajczak, Abigail C. Schmidt

**Affiliations:** ^1^ Division of Biology Kansas State University Manhattan Kansas USA; ^2^ Department of Biology University of North Carolina Greensboro Greensboro North Carolina USA; ^3^ Department of Wildland Resources Utah State University Logan Utah USA

**Keywords:** alternative stable states, bush encroachment, disturbance, extreme fire, hysteresis, LiDAR, regime shifts, woody expansion

## Abstract

Woody encroachment is a pervasive threat in grasslands worldwide. Encroachment has proven resilient to many nature‐based interventions, including frequent burning and destocking grazers. A potential solution is high‐intensity fires—or “extreme” fires—which combine high fuel loads with burning under dry windy conditions. Large‐scale tests of high‐intensity fire are limited and have had mixed results. We compared two catchments of tallgrass prairie heavily encroached by resprouting shrubs, with similar land‐use histories, including long‐term grazer destocking. In 2021, one catchment burned under typical low‐intensity conditions. The other burned on a dry, windy day, producing a high‐intensity fire with approximately 10‐m flame lengths. Based on remote‐sensed and on‐the‐ground measurements, low‐intensity fire decreased woody encroachment initially, with nearly full recovery of shrub height/cover after 2 years. High‐intensity fire caused top‐kill across almost the entire catchment, but vigorous resprouting resulted in nearly full height recovery after 3 years and an increase in woody cover. After the high‐intensity fire, diversity metrics and forb cover increased, then fell to prefire values 1 year later. Our results suggest that high‐intensity fires may help constrain low‐severity encroachment. However, reversing encroachment will require a long‐term approach and novel combinations of interventions, especially where resprouting shrubs are expanding.

## INTRODUCTION

One of the most widespread threats to grassland conservation and ecosystem services is woody plant encroachment (WPE)—the expansion of shrubs and trees in historic grassland regions, often at the expense of native herbaceous species (Archer et al., 2017; Stevens et al., 2017). WPE typically reduces streamflow (Keen et al., 2023), habitat for grassland species (Archer et al., 2017), and forage for livestock (Morford et al., 2022). WPE has proven difficult to reverse in mesic grasslands and similar systems, because once established, encroaching shrubs become resilient to fire (Fogarty et al., 2025), creating the need for strategies that reverse WPE while minimizing unintended consequences.

Multiple global change drivers are simultaneously favoring WPE. At local scales, fire suppression or heavy grazing are common in many grasslands and are a proximate cause of WPE (Archer et al., 2017). In some grasslands, increasing winter precipitation and precipitation intensity result in greater soil water availability in deep soils, where woody plants have an advantage over most grasses (Miller et al., 2010). At the global scale, elevated atmospheric CO_2_ is stimulating the growth and drought resistance of C_3_ shrubs more than competing C_4_ grasses, favoring WPE (Bond & Midgley, 2012a). For the foreseeable future, these large‐scale factors will likely reduce the effectiveness of disturbance regimes and extreme droughts that historically prevented WPE (Bond & Midgley, 2012a; Keen et al., 2024).

As WPE intensifies, changes in shrub demography and key feedback mechanisms make reversing encroachment increasingly difficult (Ratajczak et al., 2014). The dense canopy of encroaching shrubs drastically reduces grasses, replacing flammable grass fuels with less flammable shrub leaves (Archer et al., 2017). In tallgrass prairies, a single shrub can form >50 m^2^ thickets (Briggs et al., 2005), with 75% less fine fuel mass in the shrub center (Ratajczak et al., 2011). Well‐established shrubs have thicker bark and taller apical meristems that provide fire protection (Bond & Midgley, 2012b). If fires overcome these barriers and cause top‐kill of stems, mature shrubs can tap into belowground carbon stores and resprout vigorously (Briggs et al., 2005). After decades of encroachment, the functional diversity of woody species increases, potentially creating a portfolio effect that enhances the shrub community's resilience to changing conditions (Wedel et al., 2025). This suite of mechanisms makes shrub encroachment resilient to many management interventions, including frequent low‐intensity prescribed fires (Collins et al., 2021; Fogarty et al., 2025). The example of WPE closely matches what Standish et al. (2014) termed *unhelpful resilience*, where transitions to undesirable ecological states become difficult to reverse (e.g., desertification, spread of macroalgae in coral reefs).

Intentional use of high‐intensity fires, sometimes referred to as extreme fire, is a potential strategy to overcome the unhelpful resilience of shrub encroachment. High‐intensity fires occur when large fuel loads are burned during dry and windy conditions; the result is fires with a large release of energy, rapid spread, and (often) erratic behavior, such as long‐distance spotting (Archibald, 2016; Keeley, 2009; Twidwell et al., 2021). High‐intensity fires can increase woody mortality by burning hot enough to scorch the shrub cambium and ignite woody fuels (Smit et al., 2010). However, not all high‐intensity fires have proven effective, and many required follow‐up interventions (Fogarty et al., 2025; Smit et al., 2016; Strydom et al., 2023). Most experiments on high‐intensity fires have been confined to small‐scale plots (Scholtz et al., 2022; Twidwell et al., 2016; but see Donovan et al., 2025). Observational studies on accidental high‐intensity fires have provided important insights but often lack nearby “control” fires conducted under typical prescribed conditions (e.g., portions of the Starbuck fire in Kansas, USA; Parker et al., 2022). Finally, most studies on high‐intensity fire have focused on non‐resprouting trees (Donovan et al., 2025; Smit et al., 2010; Strydom et al., 2023; but see Twidwell et al., 2016), yet resprouting shrubs generally present the more challenging form of encroachment to reverse (Nippert et al., 2021).

High‐intensity fires can also have unintended consequences. High‐intensity fires have safety concerns, as high wind speeds and low relative humidity can lead to more unpredictable fire behavior. Unlike most low‐intensity fires (Hiers et al., 2025), high‐intensity fires sometimes burn hot enough to kill grass buds, decreasing grass cover and native plant diversity (Ratajczak et al., 2019; but see Donovan et al., 2025). High‐intensity fires can also cause nontarget mortality of tall trees, such as mature savanna trees and riparian vegetation, which provide beneficial ecosystem services in certain grasslands and savannas (Smit et al., 2016).

We assessed the effectiveness of high‐intensity fire to reverse WPE in a mesic grassland by comparing the effects of high‐intensity wildfire to a low‐intensity prescribed fire. Each fire took place in an approximately 53‐ha catchment (Figure 1A), which is similar to the scale that pastures are often designated in the region (colloquially, a “quarter section”). Both catchments had been destocked of grazers for decades. In 2021, one catchment burned in an accidental wildfire, with characteristics of a high‐intensity fire including long flame lengths (~10 m). The other catchment burned in the same year under typical low‐intensity conditions. We hypothesized that high‐intensity fire would lead to greater shrub top‐kill followed by a decrease in shrub cover. However, we expected that high‐intensity fire might damage the herbaceous community. Answering these questions is critical, as land stewards balance the potential risks of conducting high‐intensity fires against the costs of persistent woody encroachment.

**FIGURE 1:**
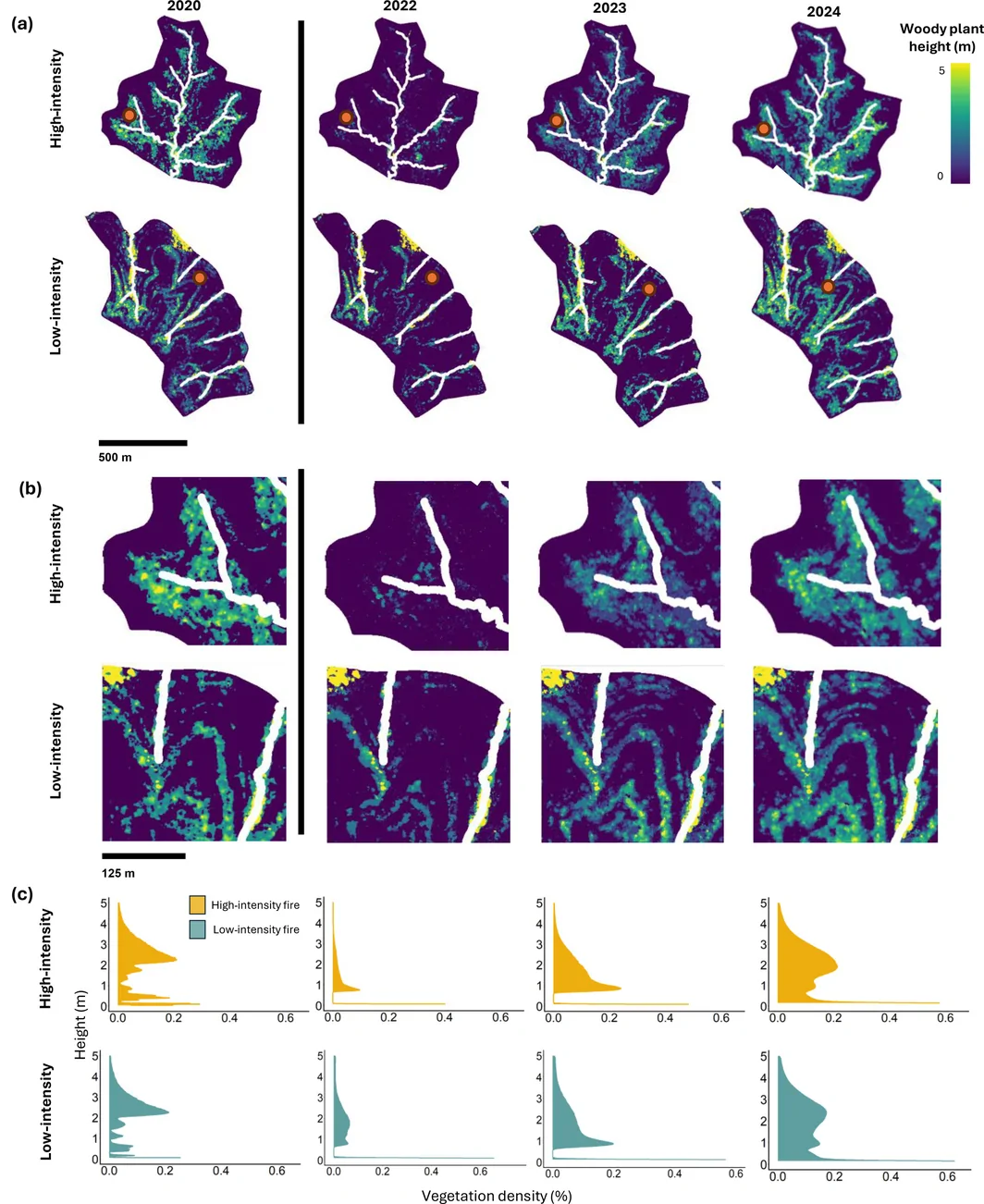
Maps of remote‐sensed woody plant cover. Panel (a) shows catchment‐scale maps before and after a high‐intensity (top) or low‐intensity fire (bottom). Orange dots denote the location of the fine‐scale portions of panel (b). Green/yellow areas show the density of woody plants over 0.95 m in height. The black vertical line denotes the fire events that occurred in spring 2021. Panel (b) shows portions of the two catchments at a finer scale. Panel (c) displays figures of vegetation density by height of vegetation throughout the years.

## METHODS

### Study site description

Konza Prairie Biological Station (KPBS) is a 3478‐ha unplowed native grassland in the Flint Hills of NE Kansas (USA)—the largest remaining region of unplowed tallgrass prairie. KPBS is divided into catchments with differing fire and grazing treatments. Most fires have been conducted in the spring under low‐intensity conditions typical of prescribed fires in the region (e.g., moderate humidity and wind speeds). KPBS has rugged topography and silty clayey loam soils that range from <50 cm deep in uplands to >1 m in lowlands. The climate is continental, with warm/wet summers and cool/dry winters. The annual climate averages are 835 mm for precipitation and 12.8°C for temperature. C_4_ perennial grasses are dominant (e.g., *Andropogon gerardii*), but forbs are also a prominent component of the plant community. In the absence of fire, WPE occurs, typically by trees and large clonal shrubs such as *Cornus drummondii*, *Prunus americana*, and *Rhus glabra* (Wedel et al., 2025).

### Fire treatments

We used catchments 4B and K4A, which we refer to as “high‐intensity” and “low‐intensity,” respectively. We began measuring WPE in these catchments in 2020 as part of another project. The catchments have nearly identical sizes (54.2 and 53.0 ha), and each has a long‐term history of grazer exclusion since 1980. The catchments have similar topography and as of 2020, levels of WPE. For 20 years, their fire regimes have been nearly synchronous, burning every 3– 4 years (Appendix S1: Figure S1, Table S1). Both catchments were scheduled to burn in 2021, but on April 3, 2021, an accidental wildfire burned 4B. Conditions included low relative humidity of 41%, average wind speeds of 6 m/s that gusted to 11 m/s, and approximately 17°C temperatures (Appendix S1: Figure S1). Flame lengths were estimated to reach 10 m by wildland fire experts who responded to the fire. These conditions and fire behavior are all consistent with a high‐intensity fire (Twidwell et al., 2016) and far outside of the usual parameters for low‐intensity prescribed fires in the region. The low‐intensity fire in catchment K4A was conducted on April 12, 2021, under high relative humidity and low temperature, typical of a low‐intensity prescribed fire (Appendix S1: Figure S1). No additional fires or management occurred on these catchments after the fires in 2021.

### Airborne LiDAR measurements

To measure catchment‐scale WPE, we used Light Detection and Ranging (LiDAR) from the National Science Foundation (NSF) National Earth Observatory Network (NEON) Aerial Observation Platform (AOP) (NEON, 2025). LiDAR‐based canopy height is one of the more effective inputs for detecting shrubs at KPBS (Noble & Ratajczak, 2025). Most years, NEON performs a flyover of KPBS using LiDAR and hyperspectral sensors mounted to a low‐flying plane. A Riegl Q780 captured the LiDAR data in 2020 and 2024. An Optech Gemini sensor was used in 2022 and 2023. The Reigl sensor yields more returns per square meter, but we have included data from both sensors for completeness. Flyovers occurred on July 7, 2020, June 13, 2022, May 26, 2023, and June 5, 2024. Deviations in timing were the result of a global pandemic in 2020 and other logistical considerations. Note that all mission plans for aerial observation were part of multiagency efforts and coordinated through NEON.

 We used “ecosystem structure,” which calculates canopy height as the difference between the height of the top LiDAR return and lowest return, per 1 x 1 m pixel (NEON, 2025). LiDAR imagery pixels with a height ≥0.95 m were considered woody, while pixels <0.95 m height were considered herbaceous dominated. Due to the timing of the flyovers, the herbaceous and woody layers were mostly two distinct heights allowing us to use these cutoffs. We also report maps and density plots of canopy height for each catchment for each year. We estimated shrub height by calculating the median value of all pixels with a height >0.95 m.

### Woody plant transects

Starting in 2020, we established 12 plots across similar topography (avoiding gullies) and soil types (deeper lowland soils typical of the Tully series) in each catchment. Each plot consisted of thirty 0.5 m × 0.5 m subplots, within which we measured the diameter of all shrub stems (see Appendix S1: Table S2). We used allometric equations to convert stem diameters into stem density, height, and leaf area (Wedel et al., 2025).

### Plant community responses to high‐intensity fire

We used data from twenty 10 m^2^ permanent plant community plots in the high‐intensity fire catchment (see Appendix S1), allowing us to calculate plant diversity (species richness and Shannon's index) and functional group cover (grasses, forbs, woody plants, and non‐native species). Unfortunately, no similar data were available from the low‐intensity fire catchment. For completeness, we also calculated the species richness of annual versus perennials and forbs versus grasses (reported in Appendix S1: Figure S2).

### Statistics

For all ground‐based measurements—woody plant transects and plant composition plots—we used mixed model ANOVAs with plot as a random effect followed by Tukey's honest‐squared difference tests, when appropriate (see Appendix S1). To analyze changes in the aerial measurements, we calculated annual woody % cover and median shrub height using LiDAR.

## RESULTS

### Remote‐sensed WPE

In 2020, the high‐intensity fire catchment had 20% woody cover, which decreased to 7% cover in 2022 postfire, then increased to 21% in 2023 and 30% in 2024 (Figure 1). Woody cover in the low‐intensity treatment was 24% in 2020, decreased to 19% in 2022, then increased to 26% in 2023, and 32% in 2024. Median shrub heights in the high‐intensity fire catchment were 2.5 m in 2020, 1.8 m in 2022 and 2023, and 2.2 m in 2024. With low‐intensity fire, median shrub heights were 3.0 m in 2020, 3.5 m in 2022, 2.6 m in 2023, and 2.7 m in 2024.

### Woody plant transects

In 2020, differences in stem density and height, between catchments, were highly insignificant (*p* = 0.99; Figure 2A; Appendix S1: Tables S3–S5). In 2022, the mean shrub height in the high‐intensity fire catchment was significantly lower than all other pairwise comparisons but recovered to prefire values in 2023 (*p* = 0.0012, Figure 2A; Appendix S1: Tables S3–S5). Postfire, average height temporarily increased in the low‐intensity fire, likely reflecting higher mortality of small stems and persistence of large stems. There were no significant changes in stem density or leaf area across treatments and years (Figure 2B,C; Appendix S1: Tables S3–S5).

**FIGURE 2:**
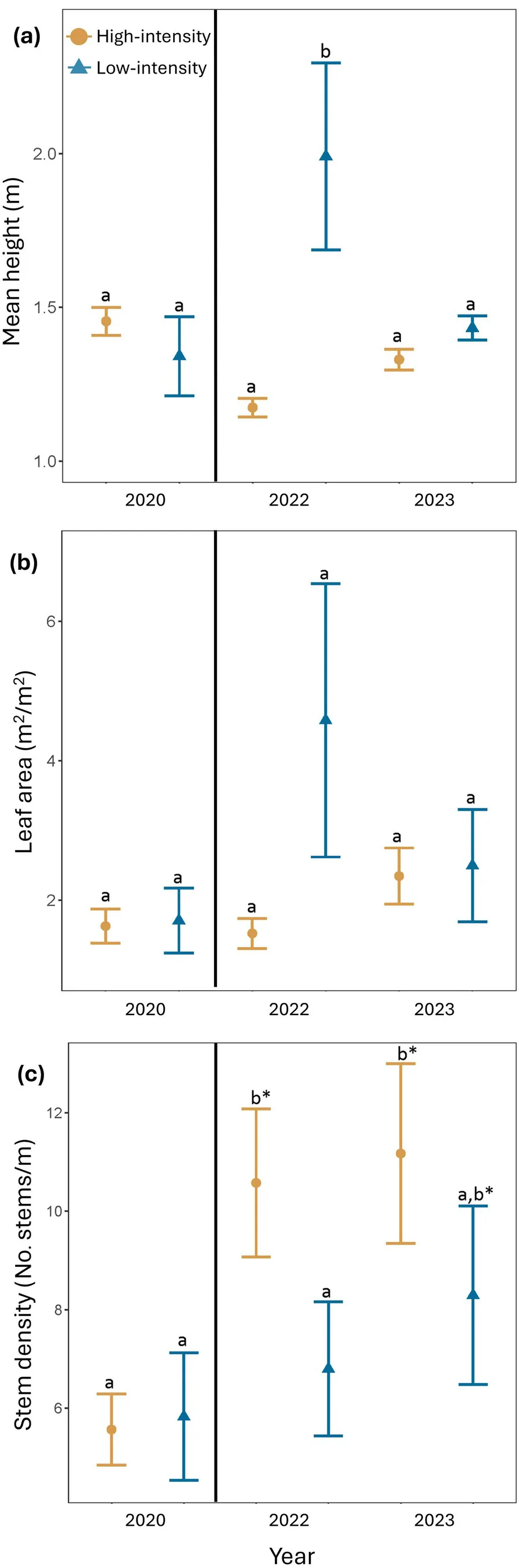
Changes in on‐the‐ground plot measurements of (a) mean height, (b) leaf area, and (c) stem density of woody species before and after a high‐intensity (orange) or low‐intensity (blue) fire treatment. Points show plot means and error bars denote SEM. The black vertical line represents the 2021 fire event. Letters represent significance from post hoc tests, with asterisks representing marginal significance.

### Herbaceous plant community

Plant community data were only available for the high‐intensity fire catchment. From 2020 to 2024, grass and woody plant cover did not change significantly (Figure 3; Appendix S1: Table S6). After high‐intensity fire, plant species richness increased by approximately 50%, Shannon diversity by approximately 30%, and forb cover by approximately 70%. All metrics fell to prefire values 1 year later (Figure 3). Much of the temporary increase in species richness was due to annuals and forb species (Appendix S1: Figure S2).

**FIGURE 3:**
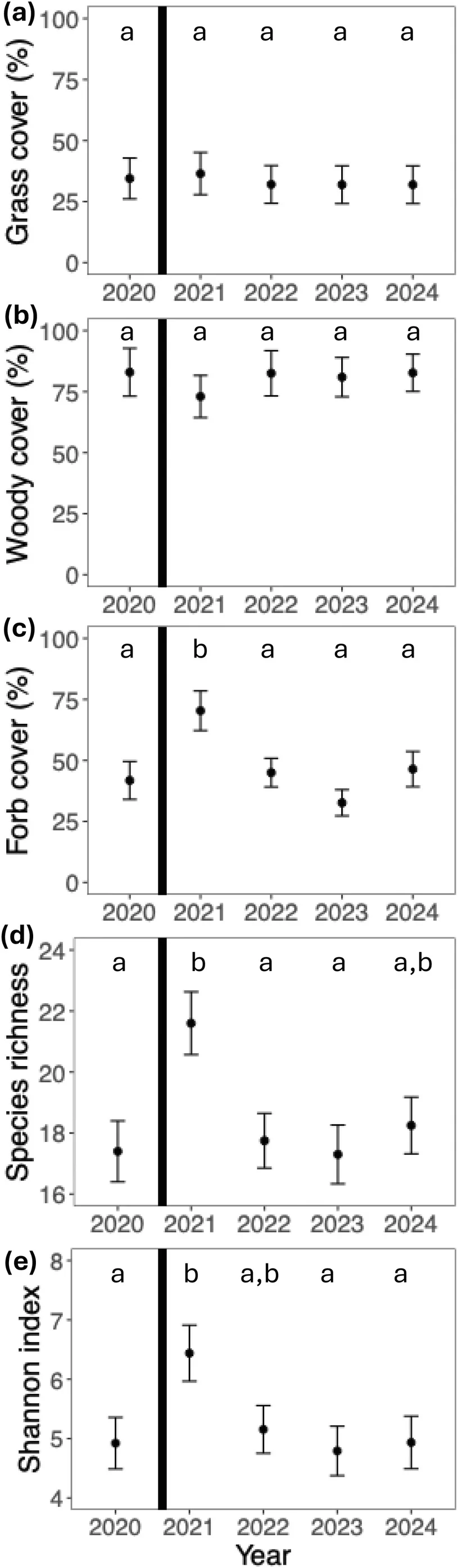
Changes in plant communities in the high‐intensity fire treatment: (a) grass cover, (b) forb cover, (c) woody cover, (d) species richness per 10‐m^2^ plot, and (e) Shannon diversity index, expressed as the exponent of Shannon's index. The black vertical line denotes the 2021 fire event. Lowercase letters denote significant differences between years. Note that cover values can exceed 100% for functional groups because the plant community data have units of each species percent canopy cover per unit ground area, and these communities have overlapping canopies.

## DISCUSSION

We addressed whether high‐intensity, sometimes referred to as extreme fire, could begin the process of reversing WPE, at scale. High‐intensity fire led to initial declines in WPE that far exceeded a typical low‐intensity fire. However, this success was short‐lived. Two years after the high‐intensity fire, we found nearly complete recovery by encroaching shrubs across most metrics. Three years after the high‐intensity fire, shrub cover was higher than prefire measurements and demographic recovery was robust, with a near doubling of shrub stem density compared to prefire values. Our results suggest that a single high‐intensity fire was able to overcome some of the encroaching shrubs' resistance but not their ecological resilience (sensu Standish et al., 2014; Walker et al., 2004). Therefore, further interventions are needed. Our results and other studies point to several promising avenues.

High‐intensity fires have successfully decreased WPE in some grasslands and savannas (Bielski et al., 2021; Twidwell et al., 2021). In North America, examples with high success often involve non‐resprouting trees, such as *Juniperus* species (Scholtz et al., 2022; but see Fogarty et al., 2025). When used under dry windy conditions, high‐intensity fires sometimes achieve >85% mortality (Scholtz et al., 2022; Twidwell et al., 2009; Twidwell et al., 2016). However, at KPBS and other mesic grasslands, the primary encroachers are clonal, resprouting shrubs (Ratajczak et al., 2011; Wedel et al., 2025). These shrubs create a zone with lower fine fuels that decreases fire intensity, limiting both the chance of top‐kill and the ability of fire to penetrate belowground and fully kill shrubs. This could explain why we did not see outright shrub mortality in this study. Clonal shrubs also have well‐protected belowground buds and carbon stores, allowing them to resprout vigorously (O'Connor et al., 2020), which could account for why we found such high resilience and the increase in stem density (Figure 1). For instance, robust resprouting and recovery by clonal shrubs has occurred despite fire combined with experimental and natural droughts (Archer et al., 2017; Keen et al., 2024).

This study reinforces arguments that reversing shrub encroachment with fire will need to focus on targeting key mechanisms—specifically opening the shrub canopy and wearing down shrubs' belowground carbon stores. Shrub canopies can impose severe light limitation (Tooley et al., 2024), slowing the production of herbaceous fine fuels. Fire can thin shrub canopies, especially when they achieve top‐kill. For instance, a study in Texas, USA, found a decline in shrub crown area and 35%–55% mortality of resprouting shrubs, stemming from a combination of high‐intensity fire during drought, followed by a low‐intensity fire (Twidwell et al., 2016). The period of lower shrub height after the high‐intensity fire might have allowed for increased grass growth due to an increase in light availability and a decrease in shrub dominance, leading to a higher abundance of fine fuels inside of shrub perimeters. Therefore, in our study, a high‐intensity fire followed by low‐intensity fire between the years 2022 and 2023 may have been more successful.

Once fuels and fire intensity increase enough to kill aboveground shrub stems, there is still the matter of reducing belowground carbon reserves enough to cause mortality. Another study at KPBS, also in a destocked catchment, burned and top‐killed shrub encroached areas annually for 20 years, but found no decline in shrub area, suggesting shrubs have yet to exhaust their carbon reserves (Collins et al., 2021). Yet, in just 3 years, a nearby study applying artificial browsing reduced shrub starch stores by 50% and following fire, saw a 90% reduction in shrub cover (O'Connor et al., 2020). Measurements of belowground tissues strongly suggest that a depletion of carbon reserves was the causal mechanism in the study with artificial browsing (O'Connor et al., 2020). Native mixed feeders or browsers, especially larger species such as elk (*Cervus canadensis*), could have similar effects. Similar strategies have been effective in African grasslands and savannas (Capozzelli et al., 2020), as have applications with domesticated browsers (e.g., goats, Elias & Tischew, 2016). Although we note that sometimes browsing effects are offset if herbivore diets include substantial amounts of herbaceous plants, which are also a source of fine fuels (Conard & Gipson, 2012).

Another appeal of combining fire with other interventions is that high‐intensity fires can have negative effects. In one African savanna, high‐intensity fire increased mortality of non‐encroaching tree species that are essential parts of the ecosystem (Smit et al., 2016). High‐intensity fires, including one that affected our site in 2011, can cause a decrease in grass cover and a lasting increase in weedy annual forbs (see Ratajczak et al., 2019). Fortunately, we did not find that high‐intensity fire reduced native plant diversity or grass cover, but we also did not see a lasting rise in native plant diversity, which has occurred elsewhere (as in Donovan et al., 2025). Although we did observe that the high‐intensity fire caused a temporary increase in forbs and annual species, it had no lasting impacts on the herbaceous community (Figure 3; Appendix S1: Figure S2). This suggests that there is leeway to increase fire intensity in future fires.

### Conclusion

Our results match a broader observation in environmental science, that many undesirable state changes are nonetheless resilient (Standish et al., 2014). Given the widespread and acute effects of WPE, there is a natural desire for “cure‐all” solutions that quickly reverse shrub abundance and foster a rapid return of the herbaceous community. That was certainly the case here. We were optimistic following the 2021 wildfire, because we had never observed such widespread top‐kill of shrubs on site. Following the initial wildfire with another prescribed burn, the subsequent year may have led to increased woody mortality and further reversal of WPE on site. This study and many others suggest that fully reversing shrub encroachment, especially with interventions that mimic natural processes, will be costly, complex, and time‐consuming (Nippert et al., 2026). In many instances, it took decades for WPE to unfold, and we might expect fully reversing WPE to take just as long. Our results support tenets of the precautionary principle that preventing WPE in early stages will often be more effective than attempting to reverse severe WPE. Given the effects on well‐established WPE, high‐intensity fires may help slow or halt WPE in areas where shrub expansion is less severe or yet to occur.

## AUTHOR CONTRIBUTIONS

Kalea R. Nippert‐Churchman and Zak Ratajczak conceived the ideas, designed the study, and analyzed the data. Kalea R. Nippert‐Churchman, Zak Ratajczak, and Abigail C. Schmidt collected the data. Kalea R. Nippert‐Churchman led writing, with support from Zak Ratajczak.

## CONFLICT OF INTEREST STATEMENT

The authors declare no conflicts of interest.

## Supporting information


Appendix S1.

## Data Availability

Data are available as follows: fire effect data (Nippert‐Churchman, 2026) is available in Zenodo at https://doi.org/10.5281/zenodo.19863348; plant species composition data (Hartnett et al., 2025) and meteorological data (Nippert, 2025) are available in the Environmental Data Initiative's Data Portal at https://doi.org/10.6073/pasta/c2dde97352fb0e25ab749765967997b9 and https://doi.org/10.6073/pasta/a267f9b0995f6fa91340ba5886ee2273, respectively; ecosystem structure data are provided by NEON (National Ecological Observatory Network) at https://data.neonscience.org/data-products/DP3.30015.001.
