# Supplementary material for: High‐intensity fire and long‐term destocking causes top kill but fails to reverse encroachment and restore biodiversity
Source: Ecol Appl. 2026 Oct 22;36(7):e70341. doi: 10.1002/eap.70341 (PMC13689527; doi:10.1002/eap.70341)
Supplement: Supplementary file 1 [file EAP-36-e70341-s001.pdf]

Appendix S1

High-intensity fire and long-term destocking causes top kill but fails to reverse encroachment and restore biodiversity

Kalea R. Nippert-Churchman, Zak Ratajczak, Abigail C. Schmidt

Ecological Applications

Section S1. Methods and detailed results for woody plant transects

Woody transect data collection: We placed plots with double randomization and at least 20 m between the edge of each plot. From the center of each plot, we established three 20 m transects, with a common centroid and 120º of rotation between transects. Along each transect, we established 0.5 x 0.5 m subplots at 2 m intervals, resulting in 30 subplots per plot. In each sub-plot, we measured the species for each stem at least 0.5 m tall and used digital calipers to measure stem diameter at 0.5 m height. We repeated measurements in 2022 and 2023 to determine changes in WPE. All sampling occurred over 2 weeks each June.

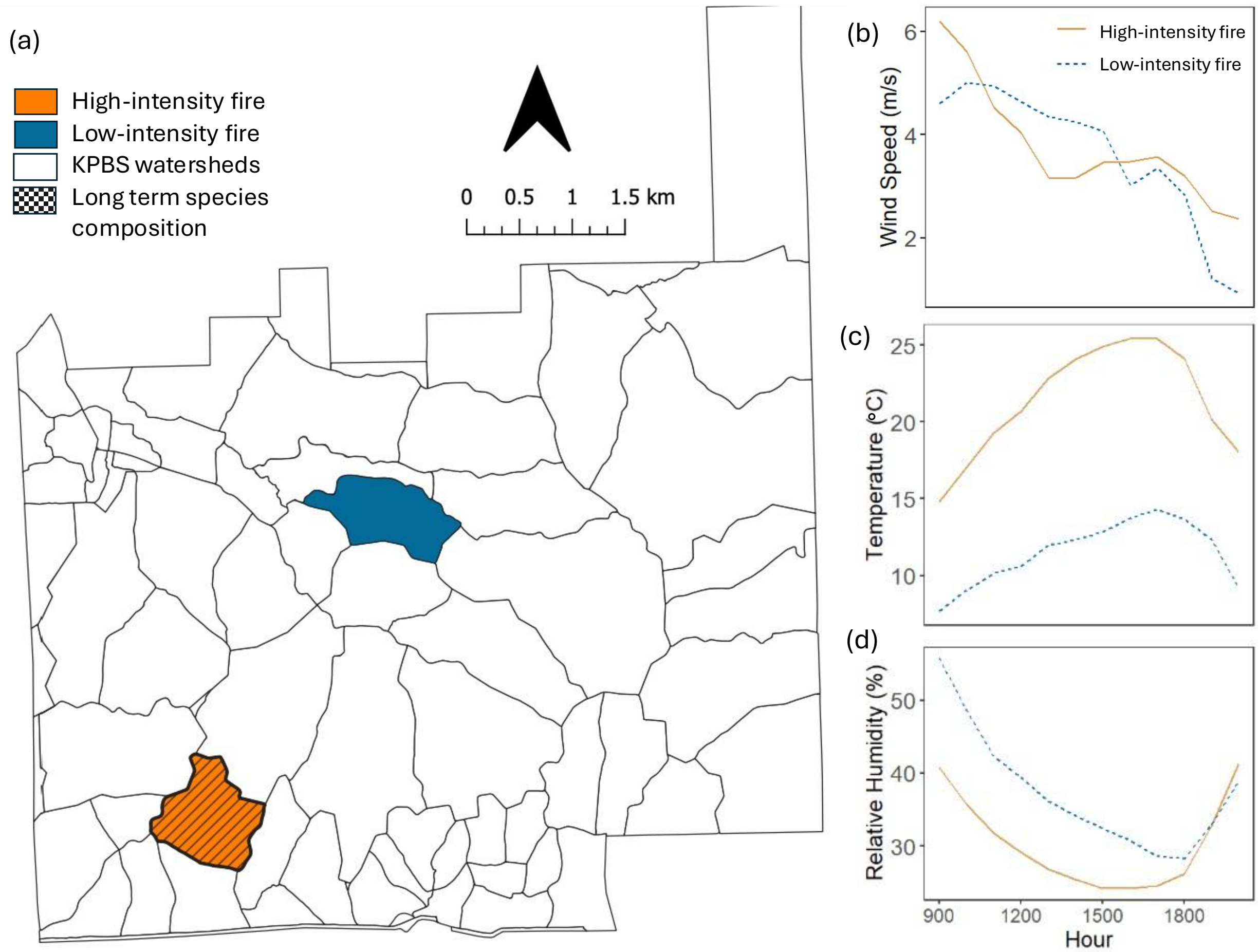

Figure S1. Map of the study area (a), and weather conditions on days of both burns (b, c, d). The high-intensity treatment is denoted in dark orange. The low-intensity treament is denoted in blue. Black hatching represents long-term species composition plots. Graphs of hourly (b) wind speed, (c) temperature, and (d) relative humidity for the days of the high-intensity and low-intensity fires in April 2021. The high-intensity fire is denoted with a solid orange line, while the low-intensity fire is denoted with a dashed blue line.

Table S1. Burn dates and fire type for the high-intensity and low-intensity catchments over the last 20 years. Both catchments have burned every four years since 1980. The 2025 fires were conducted after the conclusion of this study.

**High-intensity (4b)**		**Low-intensity (K4a)**	
Date	Fire Type	Date	Fire Type
4/15/2005	Prescribed	4/14/2005	Prescribed
4/22/2009	Prescribed	3/17/2009	Prescribed
4/13/2013	Prescribed	3/15/2003	Prescribed
4/13/2017	Prescribed	3/9/2017	Prescribed
4/3/2021	Wildfire	4/12/2021	Prescribed
4/21/2025	Prescribed	3/27/2025	Prescribed

Table S2. Summary table of all data types collected over each year of the study

**Measurement**	**Catchment**	**2020**	**2021**	**2022**	**2023**	**2024**
**Ground-based shrub density and diameter**	Low-intensity	■		■	■	
	High-intensity	■		■	■	
**LiDAR flyover with Optech Gemini sensor**	Low-intensity			■	■	
	High-intensity			■	■	
**LiDAR flyover with Riegl Q780 sensor**	Low-intensity	■				■
	High-intensity	■				■
**Long-term plant comp. data**	Low-intensity					
	High-intensity	■	■	■	■	■

Konza fire data availability:

Blair, J. and P. O'Neal. 2025. KFH01 Konza prairie fire history ver 23. Environmental Data Initiative. https://doi.org/10.6073/pasta/f7a0875273b934171aa7fd2cf4dd5ef3

Section S2. Methods for long-term data collection

Long-term Data Collection: To supplement our transect-based data and LiDAR, we leveraged long-term plant community composition plots located in the high-intensity fire catchment to assess if the high-intensity fire had unintended consequences on herbaceous plant diversity and mortality. These are comprised of four 50 m long transects, with a 10 m^2 plot located at each 10 m interval along each transect, yielding a total of 20 plots. These plots are visited twice per year, once in the early growing season and once in the late growing season. The long growing season in our study region allows many species to have periods of the year when they reach peak cover. For instance, a large cohort of annuals reach peak cover in the spring and many tall perennial forb species reach peak cover late in the growing season. Therefore, for species present at both sampling periods, we use the highest value measured per plot per year, following most work with this dataset. All plots are located in lowland soils of the Tulley series. The approximate canopy cover of all vascular plants rooted within the plots is recorded using a modified Daubenmire cover class system (Hartnett et al., 2025). We used this dataset to calculate grass cover, woody cover, forb cover, species richness, and Shannon’s Index.

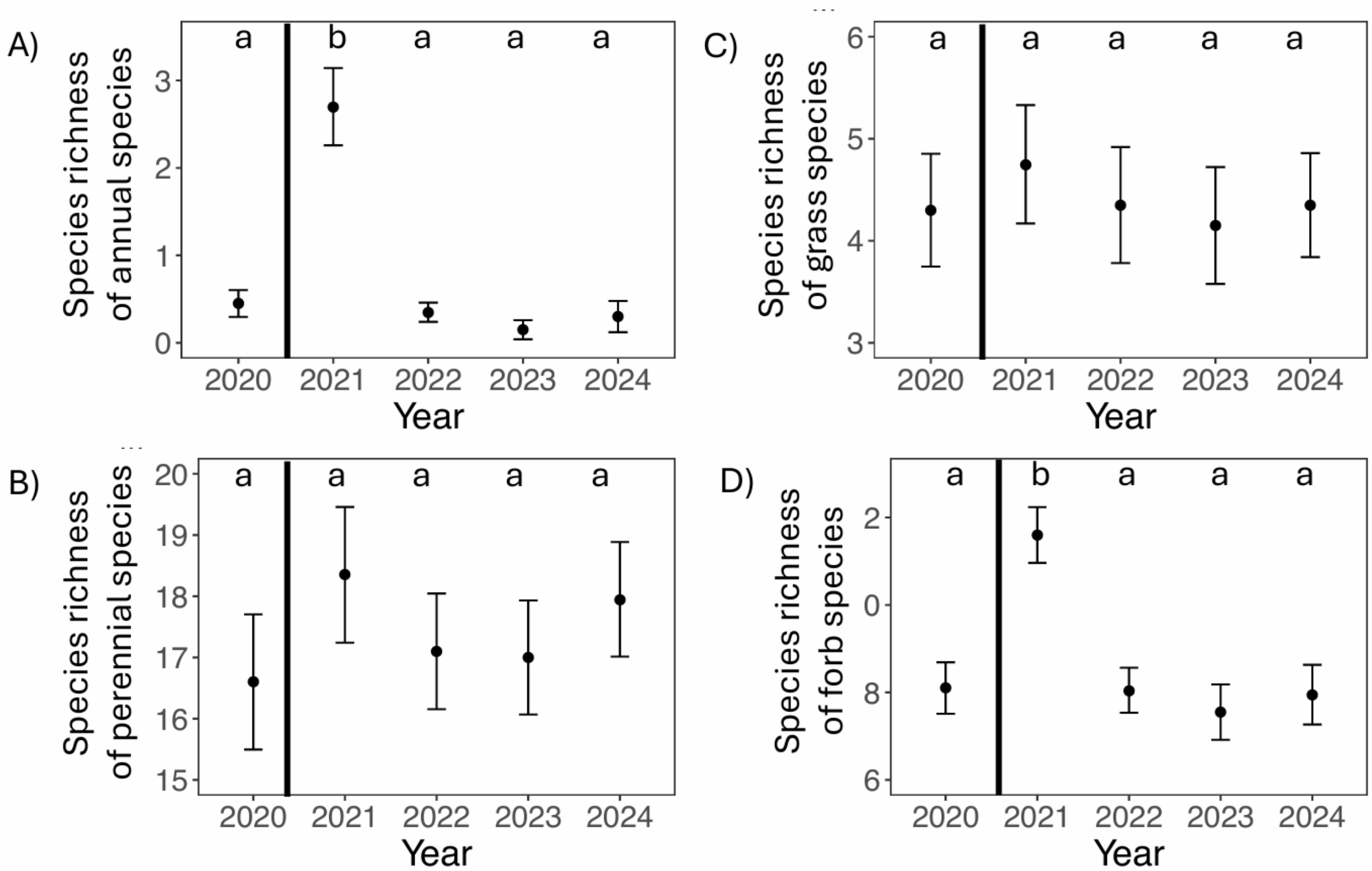

Figure S2. Changes in species richness of annual species (a), perennial species (b), grass species (c), and forb species (d) from 2020 to 2024 in the high-intensity fire catchment.

Long-term Species composition data availability:

Hartnett, D. C., Collins, S. L., Ratajczak, Z. 2025. PVC02 Plant species composition on selected watersheds at Konza Prairie. Environmental Data Initiative. http://dx.doi.org/10.6073/pasta/c2dde97352fb0e25ab749765967997b9.

Section S3. Methods for data analysis

All data was analyzed using R version 4.4.1 (R Core Team, 2024).

Woody transect data analysis: Woody plant transects were grouped by plot for all analyses, with 12 plot replications in both the high-intensity and low-intensity fire treatments. Catchment and year were main effects, and plot was used as a random effect, with the interaction between both

effects included in the model. When results were significant, we performed a post-hoc Tukey's honest square difference test to explore all pairwise comparisons (using the eemeans package, Lenth, 2024). All ANOVAs and post-hoc test results are listed in tables S3, S4, and S5.

Long-term Data Analysis: Long-term plant composition data and species richness analyses were analyzed based on year, with 2020 occurring before the fire event, and 2021 – 2024 showing changes in plant community composition after the high-intensity fire. ANOVAs were performed using year as the main effect term and transect as a random effect to account for repeated sampling effects. If the main effect was significant, a post-hoc Tukey's honest square difference analysis was performed. No transformations for normality were needed. All ANOVAs and post-hoc test results are listed in tables S6, S7, and S8 for long-term community composition analysis and tables S9, S10, and S11 for species richness analysis.

References:

Lenth, R.V. (2024). *emmeans: Estimated Marginal Means, aka Least-Squares Means* (1.10.3). https://CRAN.R-project.org/package=emmeans

R Core Team. (2024). *R: A Language and Environment for Statistical Computing* (R.4.4.1). R Foundation for Statistical Computing. https://www.R-project.org

Table S3. Effects of fire treatment (catchment, *C*) and year (*Yr)* on woody plant stem density, mean height, and leaf area from two-way ANOVAs. Data from 2020 to 2023 for high-intensity and low-intensity catchments woody transect data. F-values and p-values are shown. Significant differences ($p \leq 0.05$) are in bold.

Variable	Stem density			Height			Leaf area		
	df	*F* value	*p* value	df	*F* value	*p* value	df	*F* value	*p* value
Catchment	1	3.16	0.07	1	5.67	**0.020**	1	2.17	0.14
Year	2	4.07	**0.021**	1	1.30	0.27	1	1.15	0.32
C*Yr	2	1.04	0.35	2	6.21	**0.003**	2	1.73	0.18

Table S4. Results of post-hoc emmeans test on the ANOVAs for stem density, height, and leaf area with plot as a random effect. HI represents the high-intensity treatment and LI represents the low-intensity treatment.

Variable	Stem density				Height				Leaf area			
	emmean	*SE*	Lower CL	Upper CL	emmean	*df*	Lower CL	Upper CL	emmean	*df*	Lower CL	Upper CL
HI 2020	5.57	14	2.58	8.55	1.45	49	1.21	1.70	1.63	49	0.03	3.23
LI 2020	5.33	15	2.12	8.54	1.38	30	1.06	1.70	1.65	31	-0.39	3.69
HI 2022	10.58	14	7.59	13.56	1.17	49	0.93	1.42	1.52	49	-0.07	3.12
LI 2022	6.42	15	3.20	9.63	1.95	30	1.63	2.27	4.53	31	2.48	6.58
HI 2023	11.17	14	8.19	14.16	1.33	49	1.08	1.58	2.35	49	0.75	3.94
LI 2023	7.91	15	4.70	11.13	1.39	30	1.07	1.71	2.45	31	0.40	4.49

Table S5. Pairwise comparison of watershed and year for stem density, height, and leaf area with plot as a random effect. HI represents the high-intensity treatment and LI represents the low-intensity treatment.

Variable	Stem density				Height				Leaf area			
	estimate	df	t ratio	p value	estimate	df	t ratio	p value	estimate	Df	t ratio	p value
HI 2020 - LI 2020	0.23	29	0.11	1.000	0.08	54	0.39	0.999	-0.02	55	-0.02	1.000
HI 2020 - HI 2022	-5.01	43	-5.97	<0.001	0.28	43	1.62	0.592	0.10	43	0.09	1.000
HI 2020 - LI 2022	-0.85	29	-0.42	0.998	-0.49	53	-2.47	0.153	-2.90	54	-2.26	0.227
HI 2020 - HI 2023	-5.61	43	-6.67	<0.001	0.12	43	0.72	0.979	-0.72	43	-0.64	0.987
HI 2020 - LI 2023	-2.35	29	-1.14	0.859	0.07	53	0.34	0.999	-0.82	54	-0.64	0.988
LI 2020 - HI 2022	-5.24	29	-2.56	0.142	0.20	54	1.02	0.910	0.12	55	0.10	1.000
LI 2020 - LI 2022	-1.08	44	-1.25	0.808	-0.57	45	-3.22	0.027	-2.88	45	-2.52	0.141
LI 2020 - HI 2023	-5.84	29	-2.85	0.078	0.05	54	0.24	0.999	-0.70	55	-0.55	0.994
LI 2020 - LI 2023	-2.58	44	-2.98	0.050	-0.01	45	-0.06	1.000	-0.80	45	-0.70	0.981
HI 2022 - LI 2022	4.16	29	2.02	0.351	-0.77	53	-3.87	0.004	-3.00	54	-2.34	0.195
HI 2022 – HI 2023	-0.60	43	-0.71	0.980	-0.16	43	-0.90	0.945	-0.82	43	-0.73	0.977
HI 2022 – LI 2023	2.66	29	1.30	0.785	-0.21	53	-1.07	0.891	-0.92	54	-0.72	0.979
LI 2022 – HI 2023	-4.75	29	-2.31	0.221	0.62	53	3.09	0.036	2.18	54	1.70	0.536
LI 2022 – LI 2023	-1.50	43	-1.78	0.489	0.56	43	3.22	0.028	2.08	43	1.85	0.444
HI 2023 – LI 2023	3.26	29	1.59	0.614	-0.06	53	-0.29	0.999	-0.10	54	-0.08	1.000

Table S6. Long-term plant composition ANOVA tables

Variable	Grass cover			Woody cover			Forb cover		
	df	*F* value	*p* value	df	*F* value	*p* value	df	*F* value	*p* value
Year	4	0.34	0.85	4	0.98	0.42	4	5.57	**<0.001**

Variable	Species Richness			Shannon's Index		
	df	*F* value	*p* value	df	*F* value	*p* value
Year	4	3.65	**0.008**	4	3.33	**0.014**

Table S7. Results of post-hoc emmeans test on the ANOVAs for long-term community composition data.

	Grass Cover				Woody Cover				Forb Cover			
	emmean	SE	Lower CL	Upper CL	emmean	SE	Lower CL	Upper CL	emmean	SE	Lower CL	Upper CL
2020	34.5	18.6	-24.6	93.6	83.0	19.6	20.5	145	41.8	10.9	7.1	76.5
2021	36.5	18.6	-22.6	95.6	73.0	19.6	10.6	135	70.3	10.9	35.7	105.0
2022	32.0	18.6	-27.1	91.1	82.5	19.6	20.1	145	45.0	10.9	10.3	79.7
2023	31.9	18.6	-27.2	91.0	81.0	19.6	18.5	143	32.6	10.9	-2.0	67.3
2024	31.9	18.6	-27.2	91.0	82.7	19.6	20.3	145	46.5	10.9	11.8	81.1

	Species Richness				Shannon's Index			
	emmean	SE	Lower CL	Upper CL	emmean	SE	Lower CL	Upper CL
2020	17.4	1.1	13.9	20.9	4.9	0.7	2.8	7.1
2021	21.6	1.1	18.1	25.1	6.4	0.7	4.3	8.6
2022	17.8	1.1	14.3	21.2	5.2	0.7	3.0	7.3
2023	17.3	1.1	13.8	20.8	4.8	0.7	2.6	6.9
2024	18.2	1.1	14.8	21.7	4.9	0.7	2.8	7.1

Table S8. Pairwise comparison of years for long-term species composition data.

Variable	Grass Cover				Woody Cover				Forb Cover			
	estimate	df	t ratio	p value	estimate	df	t ratio	p value	estimate	df	t ratio	p value
2020 – 2021	-2.00	92	-0.40	0.994	9.95	92	1.65	0.470	-28.55	92	-3.41	0.009
2020 – 2022	2.45	92	0.49	0.988	0.43	92	0.07	1.000	-3.20	92	-0.38	0.995
2020 – 2023	2.55	92	0.51	0.986	2.00	92	0.33	0.997	9.15	92	1.09	0.810
2020 – 2024	2.55	92	0.51	0.986	0.23	92	0.04	1.000	-4.65	92	-0.56	0.981
2021 – 2022	4.45	92	0.90	0.898	-9.53	92	-1.58	0.514	25.35	92	3.03	0.026
2021 – 2023	4.55	92	0.92	0.891	-7.95	92	-1.32	0.680	37.70	92	4.50	< 0.001
2021 – 2024	4.55	92	0.92	0.891	-9.73	92	-1.61	0.493	23.90	92	2.85	0.042
2022 – 2023	0.10	92	0.02	1.000	1.58	92	0.26	0.999	12.35	92	1.47	0.582
2022 – 2024	0.10	92	0.02	1.000	-0.20	92	-0.03	1.000	-1.45	92	-0.17	0.999
2023 – 2024	0.00	92	0.00	1.000	-1.78	92	-0.29	0.998	-13.80	92	-1.65	0.472

Variable	Species Richness				Shannon's Index			
	estimate	df	t ratio	p value	estimate	df	t ratio	p value
2020 – 2021	-4.20	92	-3.16	0.018	-1.52	92	-2.88	0.038
2020 – 2022	-0.35	92	-0.26	0.999	-0.23	92	-0.44	0.992
2020 – 2023	0.10	92	0.08	1.000	0.13	92	0.250	0.999
2020 – 2024	-0.85	92	-0.64	0.968	-0.01	92	-0.02	1.000
2021 – 2022	3.85	92	2.90	0.037	1.28	92	2.44	0.113
2021 – 2023	4.30	92	3.24	0.014	1.65	92	3.13	0.019
2021 – 2024	3.35	92	2.52	0.095	1.50	92	2.86	0.041
2022 – 2023	0.45	92	0.34	0.997	0.36	92	0.69	0.958
2022 – 2024	-0.50	92	-0.38	0.996	0.22	92	0.42	0.994
2023 – 2024	-0.95	92	-0.72	0.953	-01.4	92	-0.27	0.999

Table S9. Long-term plant sub-group species richness ANOVA tables

Variable	Annual species richness			Perennial species richness			Species richness of grasses		
	df	*F* value	*p* value	df	*F* value	*p* value	df	*F* value	*p* value
Year	92	22.64	<0.0001	92	0.56	<0.0001	92	0.56	<0.0001

Variable	Species Richness of forbs		
	df	*F* value	*p* value
Year	92	7.76	<0.000

Table S10. Results of post-hoc emmeans test on the ANOVAs for long-term species data for plant sub-group species richness analysis.

Variable	Annual species richness				Perennial species richness				Species richness of grasses			
	emmean	*SE*	Lower CL	Upper CL	emmean	*SE*	Lower CL	Upper CL	emmean	*SE*	Lower CL	Upper CL
2020	0.45	0.28	-0.43	1.33	16.6	1.21	12.8	20.4	4.30	0.96	1.24	7.36
2021	2.70	0.28	1.82	3.58	18.4	1.21	14.5	22.2	4.75	0.96	1.69	7.81
2022	0.35	0.28	-0.53	1.23	17.1	1.21	13.3	20.9	4.35	0.96	1.29	7.41
2023	0.15	0.28	-0.73	1.03	17.0	1.21	13.2	20.8	4.15	0.96	1.09	7.21
2024	0.30	0.28	-0.58	1.18	17.9	1.21	14.1	21.8	4.35	0.96	1.29	7.41

Variable	Species richness of forbs			
	emmean	*SE*	Lower CL	Upper CL
2020	8.10	0.70	5.87	10.33
2021	11.60	0.70	9.37	13.83
2022	8.05	0.70	5.82	10.28
2023	7.55	0.70	5.32	9.78
2024	7.95	0.70	5.72	10.18

Table S11. Pairwise comparison of years for long-term species composition data for plant sub-group species richness analysis.

Variable	Annual species richness				Perennial species richness				Species richness of grasses			
	estimate	df	t ratio	p value	estimate	df	t ratio	p value	estimate	df	t ratio	p value
2020 – 2021	-2.25	92	-7.05	<0.0001	-1.75	92	-1.28	0.704	-0.45	92	-0.72	0.952
2020 – 2022	0.10	92	0.31	0.998	-0.50	92	-0.37	0.996	-0.05	92	-0.08	1.000
2020 – 2023	0.30	92	0.94	0.880	-0.40	92	-0.29	0.998	0.15	92	0.24	0.999
2020 – 2024	0.15	92	0.47	0.990	-1.35	92	-0.99	0.860	-0.05	92	-0.08	1.000
2021 – 2022	2.35	92	7.37	<0.0001	1.25	92	0.92	0.891	0.40	92	0.64	0.968
2021 – 2023	2.55	92	7.99	<0.0001	1.35	92	0.99	0.860	0.60	92	0.96	0.872
2021 – 2024	2.40	92	7.52	<0.0001	0.40	92	0.29	0.998	0.40	92	0.64	0.968
2022 – 2023	0.20	92	0.63	0.970	0.10	92	0.07	1.000	0.20	92	0.32	0.999
2022 – 2024	0.05	92	0.16	0.999	-0.85	92	-0.62	0.971	0.00	92	0.00	1.000
2023 – 2024	-0.15	92	-0.47	0.990	-0.95	92	-0.70	0.957	-0.20	92	-0.32	0.999

Variable	Species richness of forbs			
	estimate	df	t ratio	p value
2020 – 2021	-3.50	92	-4.14	0.001
2020 – 2022	0.05	92	0.06	1.000
2020 – 2023	0.55	92	0.65	0.966
2020 – 2024	0.15	92	0.18	0.999
2021 – 2022	3.55	92	4.20	0.001
2021 – 2023	4.05	92	4.80	0.0001
2021 – 2024	3.65	92	4.32	0.0004
2022 – 2023	0.50	92	0.59	0.976
2022 – 2024	0.10	92	0.12	1.000
2023 - 2024	-0.40	92	-0.47	0.990
